# Immunotherapy in liver cancer: overcoming the tolerogenic liver microenvironment

**DOI:** 10.3389/fimmu.2024.1460282

**Published:** 2024-09-03

**Authors:** Yanju Liu, Hongyuan Yang, Tian Li, Na Zhang

**Affiliations:** ^1^ Department of Infectious Diseases, Weifang People’s Hospital, Weifang, Shandong, China; ^2^ School of Basic Medicine, Fourth Military Medical University, Xi’an, China

**Keywords:** immunotherapy, liver cancer, cancer microenvironment, combinational therapy, therapeutic advances

## Abstract

Liver cancer is a major global health concern, ranking among the top causes of cancer-related deaths worldwide. Despite advances in medical research, the prognosis for liver cancer remains poor, largely due to the inherent limitations of current therapies. Traditional treatments like surgery, radiation, and chemotherapy often fail to provide long-term remission and are associated with significant side effects. Immunotherapy has emerged as a promising avenue for cancer treatment, leveraging the body’s immune system to target and destroy cancer cells. However, its application in liver cancer has been limited. One of the primary challenges is the liver’s unique immune microenvironment, which can inhibit the effectiveness of immunotherapeutic agents. This immune microenvironment creates a barrier, leading to drug resistance and reducing the overall efficacy of treatment. Recent studies have focused on understanding the immunological landscape of liver cancer to develop strategies that can overcome these obstacles. By identifying the specific factors within the liver that contribute to immune suppression and drug resistance, researchers aim to enhance the effectiveness of immunotherapy. Prospective strategies include combining immunotherapy with other treatments, using targeted therapies to modulate the immune microenvironment, and developing new agents that can bypass or counteract the inhibitory mechanisms in the liver. These advancements hold promise for improving outcomes in liver cancer treatment.

## 1 Introduction

Neoplasms remain the main killer worldwide (1–3). Among which, liver cancer, predominantly hepatocellular carcinoma (HCC), stands as one of the leading causes of cancer-related deaths worldwide (4–9). Despite advances in oncological therapies, the prognosis for liver cancer patients remains dire, especially in cases diagnosed at advanced stages (10). Traditional treatments, such as resection, transplantation, and systemic chemotherapy, offer limited efficacy and often come with significant side effects (11). This backdrop underscores the urgent need for innovative therapeutic approaches, among which immunotherapy has emerged as a promising candidate (12).

Immunotherapy, which harnesses the body’s immune system to fight cancer, has significantly transformed the treatment of various malignancies (13–16), marking a shift from traditional therapies by focusing on the interactions between cancer cells and the immune system (17). Applying immunotherapy in liver cancer, however, poses distinct challenges, primarily due to the liver’s unique immunological characteristics (18). The liver is not only a crucial metabolic organ but also plays a significant role in immunology (19). Its specialized microenvironment, inherently inclined towards tolerance for normal functioning, paradoxically provides a protective environment for tumor cells, complicating the effectiveness of immunotherapy in liver cancer (20).

The tolerogenic nature of the liver is characterized by a distinct array of immune cells and regulatory pathways (21). This environment is adept at maintaining immune homeostasis and preventing overactive responses to the myriad of antigens constantly presented to it, primarily from the gut via the portal circulation (22). In the context of HCC, this immunological landscape facilitates immune evasion, allowing cancer cells to thrive and proliferate under the radar of immune surveillance (23, 24).

Addressing these challenges requires a deep understanding of the liver’s immune milieu and the complex interplay between tumor biology and host immunity (25). This review aims to dissect the intricacies of the liver’s immune environment and explore how current and emerging immunotherapeutic strategies are being tailored to overcome these barriers (26). We delve into the latest research underscoring the potential of immunotherapy in liver cancer (27). This review not only highlights the progress in immunotherapy but also delves into the multifaceted nature of tumor drug resistance, exploring genetic alterations, immune evasion, and the influence of the tumor microenvironment.

## 2 Immunological landscape of liver cancer

The tumor microenvironment (TME) is a complex network comprising cancer cells, immune cells, fibroblasts, endothelial cells, and the extracellular matrix, actively influencing cancer progression and response to therapies like immunotherapy (28–31). The TME supports tumor growth through angiogenesis, immune evasion, and modifying drug responses, playing a critical role in immunotherapy tolerance by mechanisms such as cytokine secretion (e.g., TGF-β, IL-10) that suppress immune responses, and the expression of checkpoint molecules like PD-L1 that help tumors evade immune detection (32). Additionally, direct interactions between tumors and immune cells can deactivate effector immune cells, contributing to the TME’s immune-suppressive nature (33). Understanding and manipulating the TME is essential for developing effective cancer therapies, combining tumor-targeting strategies with approaches to alter the TME, aiming for improved therapeutic outcomes.

**Figure 1:**
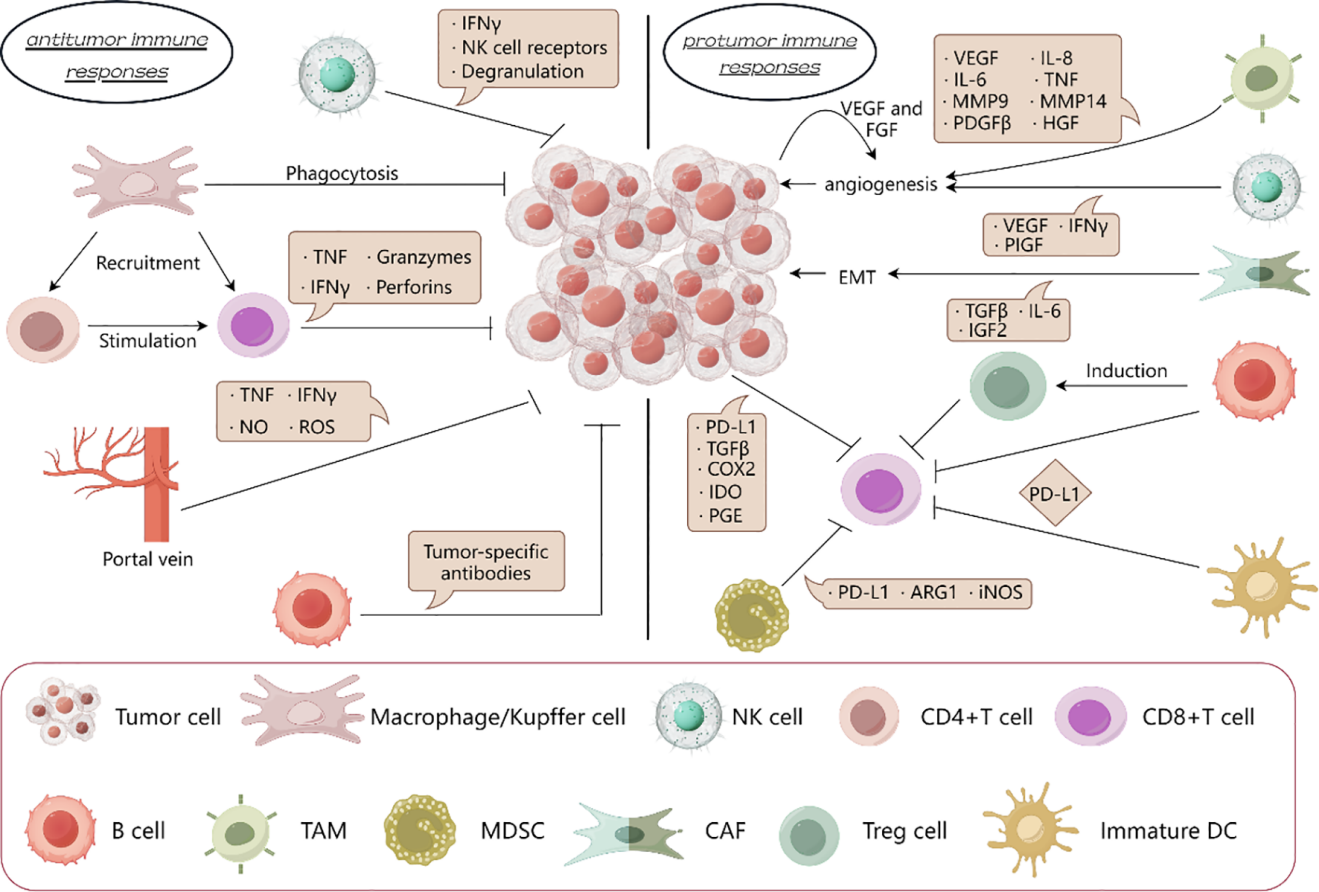
Protumor immune responses is dominant in liver cancer. In the immune microenvironment of liver cancer, the protumor immune response is superior to the anti-tumor immune response. By secreting cytokines, TAM and NK cells promote angiogenesis, CAF promotes epithelial mesenchymal transformation; Treg cells, immature DC cells, MDSCs and tumor cells suppresses the effect of CD8+T cells on tumors. And the killing effect on tumor by macrophages, NK cells and CD8+T cells is inhibited.

The liver’s immune system is uniquely adapted to its exposure to food antigens and gut-derived microbial products via the portal vein (34, 35). This exposure necessitates a predominantly tolerogenic environment to avoid an overactive immune response, which could lead to tissue damage and impaired liver function (36). The liver achieves this through a complex network of cells and signals that promote tolerance rather than immunity (37) (**Figure 1**).

### 2.1 The antigenicity of HCC

The antigenic landscape of HCC is characterized by the presence of tumor-specific antigens (TSAs) and neoantigens, essential for the immune system’s recognition and attack on cancer cells (38). While TSAs, including alpha-fetoprotein (AFP), glypican-3 (GPC3), and melanoma-associated gene 1 (MAGE-1) in HCC, are predominantly cancer-centric, their presence, albeit in lower quantities, is not exclusive to cancer cells (39). The immune system, adept at identifying abnormalities, flags these antigens, especially when overexpressed or coupled with other tumor-associated signals (40). In contrast, neoantigens, borne out of tumor-specific genetic alterations such as point mutations and chromosomal rearrangements, are exclusive to cancer cells, rendering them precise targets for immune attacks (41).

Central to immune surveillance, TSAs and neoantigens underpin various immunotherapeutic strategies for HCC, including the deployment of cancer vaccines, the transfer of adoptive T cells, and the application of checkpoint blockade therapies (42, 43). However, targeting these antigens is fraught with challenges, given the liver’s natural inclination towards immune tolerance, the heterogeneous expression of antigens across tumors, and the cancer cells’ adeptness at evading immune detection (44, 45). Moreover, the liver’s altered immune landscape, often a consequence of underlying conditions like hepatitis or cirrhosis, can significantly impact the efficacy of antigen-targeted therapies (46). Thus, delving deep into the antigenic profile of HCC, through meticulous identification and functional analysis of TSAs and neoantigens, is imperative for refining immunotherapeutic approaches and enhancing treatment precision and effectiveness against liver cancer (47).

### 2.2 Specialized immune cell populations

The liver’s immune environment is intricately composed of various specialized cell types that play pivotal roles in maintaining immune homeostasis and regulating immune responses (48). In the context of liver cancer, particularly hepatocellular carcinoma (HCC), these cells contribute to a tolerogenic milieu that can impede effective immunotherapeutic interventions (49).

Cytotoxic T Cells (CTLs) are essential for the direct killing of cancer cells. In healthy immune responses, these cells recognize and destroy cells expressing specific antigens, including tumor cells. However, in HCC, the activity of CTLs is often suppressed due to the immunosuppressive signals within the liver (50). Factors such as the upregulation of PD-L1 on tumor cells and the secretion of immunosuppressive cytokines like IL-10 and TGF-beta inhibit CTL activation and proliferation (51). Moreover, the presence of regulatory elements such as Tregs and myeloid-derived suppressor cells (MDSCs) further dampens the CTL response, allowing tumor cells to evade immune detection (52).

Regulatory T Cells (Tregs) play a critical role in maintaining immune tolerance by suppressing autoimmunity and excessive immune responses that could damage host tissues. In liver cancer, Tregs are recruited and expanded within the tumor microenvironment, where they inhibit the function of CTLs and NK cells through the secretion of suppressive cytokines like TGF-beta and IL-10 (53). This suppression helps the tumor evade immune surveillance. The enrichment of Tregs in the liver is also facilitated by the liver’s exposure to antigens from the gut, which promotes a generally tolerogenic environment (54).

As the liver’s resident macrophages, Kupffer cells are involved in clearing pathogens and cellular debris. However, in HCC, their role shifts towards promoting tumor growth and survival. They achieve this by secreting pro-tumorigenic cytokines and growth factors that enhance tumor cell proliferation, angiogenesis, and metastasis (55). Kupffer cells also contribute to the immunosuppressive environment by producing IL-10 and TGF-beta, which inhibit the functions of dendritic cells and CTLs. Additionally, they engage in crosstalk with hepatic stellate cells and cancer-associated fibroblasts to remodel the extracellular matrix, further facilitating tumor progression (56).

Dendritic Cells (DCs) are crucial for antigen presentation and the activation of T cells. However, in the liver tumor microenvironment, the function of DCs is often compromised. They are either numerically decreased or functionally impaired, which hampers their ability to present tumor antigens effectively and initiate a robust anti-tumor immune response (57). The impaired functionality of DCs in HCC is partly due to the suppressive cytokines produced by other immune cells and the tumor cells themselves.

Natural Killer (NK) and NKT Cells are important for their roles in immune surveillance and the early response to tumor formation. These cells can recognize and kill transformed cells without the need for prior sensitization to specific antigens. In liver cancer, however, their cytotoxic activity is often inhibited by the immunosuppressive cytokines in the microenvironment and by direct interactions with tumor cells that express inhibitory molecules (58).

**Figure 2:**
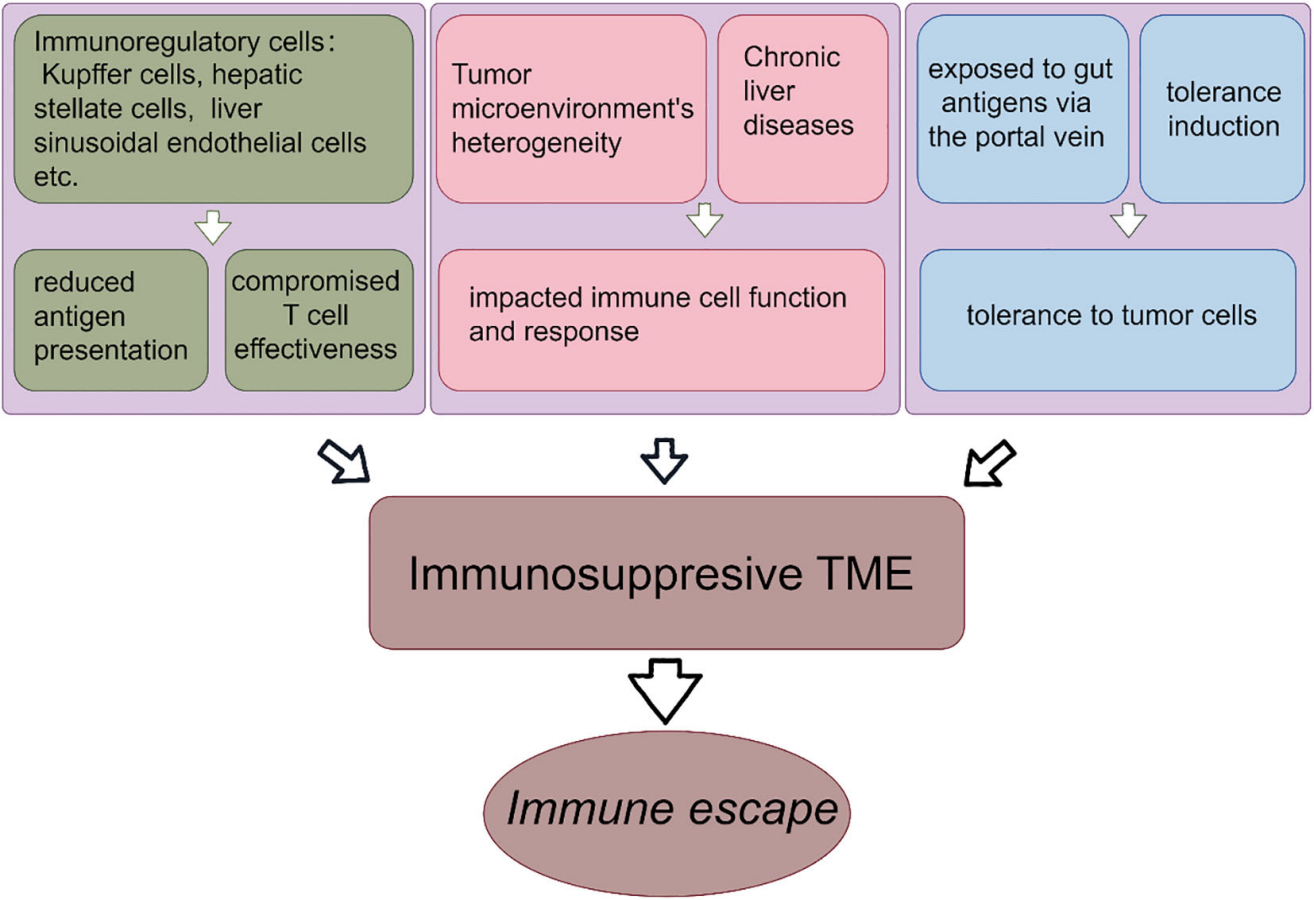
Formation of liver immunosuppressive tumor microenvironment. The formation of immunosuppressive microenvironment of liver cancer promotes the immune escape. The reduced antigen presentation by immunoregulatory cells leads to the impaired T cell effects. The heterogeneity of TME and chronic liver diseases lead to the suppression of immune function and immune response. Liver is exposed to intestinal antigens and obtains immune tolerance.

Each of these cell populations plays a significant role in the immunological landscape of liver cancer, contributing to the complexity and challenge of developing effective immunotherapeutic strategies. Understanding and manipulating the functions and interactions of these cells is key to enhancing the immune response against liver cancer (**Figure 2**).

### 2.3 Key cytokines in immune suppression

Among the cytokines that play critical roles in the liver’s immune landscape, TGF-beta and IL-10 stand out due to their potent immunosuppressive effects. TGF-beta is a multifunctional cytokine that primarily facilitates an immunosuppressive environment conducive to tumor growth and metastasis in the context of liver cancer. It acts by inhibiting the proliferation and activation of T cells and by promoting the conversion of effector T cells into regulatory T cells, thus enhancing immune tolerance (59).

IL-10, another immunosuppressive cytokine, further contributes to the complexity of the immune landscape in liver cancer by inhibiting the synthesis of pro-inflammatory cytokines, thus reducing the effectiveness of the immune response against tumor cells. It also promotes the differentiation of Tregs and hampers the antigen-presenting capabilities of dendritic cells, reducing the overall immune surveillance in HCC (60).

### 2.4 Impact of liver microenvironment on immune surveillance

The liver’s unique immunological landscape, pivotal for metabolism and detoxification, significantly shapes liver cancer immunotherapy (61). Tasked with tolerance induction, the liver, constantly exposed to gut antigens via the portal vein, distinguishes between harmful and benign substances (62). This mechanism, however, may inadvertently extend tolerance to tumor cells, complicating immunotherapy (63).

Immunoregulatory cells such as the liver sinusoidal endothelial cells, the Kupffer cells, and the hepatic stellate cells, integral to the liver’s immune tolerance, can suppress the immune response against HCC cells (64). This suppression leads to reduced antigen presentation and compromises T cell effectiveness in targeting cancer cells (65). Additionally, the liver harbors unique immune cells like NK, NKT, and γδ T cells, each with specific roles in immune surveillance, offering avenues for immunotherapy (66).

The roles of specific immune cells such as macrophages, neutrophils, and regulatory T cells (Tregs) are critical in mediating immunotherapy resistance, particularly to immune checkpoint inhibitors (ICIs). Within the liver TME, macrophages contribute significantly to ICI resistance. Their interaction with tumor cells often results in the secretion of various chemokines and cytokines that not only protect the tumor from immune attack but also enhance the recruitment of other immunosuppressive cell types. This activity establishes a feedback loop that sustains and amplifies immune suppression, diminishing the therapeutic efficacy of ICIs (67). The role of neutrophils extends beyond traditional pathogen defense to influencing the balance of the immune response in the TME. They support a suppressive environment by interacting with other immune cells and modulating their activity towards tolerance rather than immunity. Their presence in the TME correlates with poorer outcomes in immunotherapy, suggesting their potential as therapeutic targets to enhance ICI response (68). Tregs directly impact the effectiveness of ICIs by maintaining a high threshold for T cell activation. They utilize various mechanisms to suppress effector T cell function, crucially dampening the immune response against the tumor. Manipulating Treg activity or selectively reducing their numbers within the TME could potentially restore immune activity and improve responses to immunotherapies (69).

The liver tumor microenvironment’s heterogeneity, influenced by individual patient factors, affects immunotherapy’s effectiveness (70). Chronic liver diseases, often precursors to liver cancer, alter the immune landscape, impacting immune cell function and response (71).

The challenge in liver cancer immunotherapy lies in effectively activating an anti-tumor immune response without disrupting the liver’s essential tolerance mechanisms (72). Understanding the intricate balance of liver immunity is crucial for designing effective immunotherapeutic strategies (73). This involves identifying targets within the liver’s immune milieu that can be modulated to enhance the immune response against HCC cells while preserving the liver’s vital functions (74).

In conclusion, the liver’s immunological microenvironment, with its unique cellular composition and tolerance-promoting mechanisms, presents both a challenge and an opportunity for the development of effective immunotherapies for liver cancer (75). Strategies that can navigate and modulate this complex environment hold the key to successful immunotherapeutic interventions in HCC (76).

## 3 Current immunotherapeutic approaches for liver cancer

### 3.1 Genetic and molecular landscape of liver cancer

Liver cancer, particularly HCC, is characterized by distinct genetic mutations that influence both tumor behavior and interaction with the immune system. Key mutations often involve genes like TP53, known for its role in cell cycle regulation and apoptosis, and CTNNB1, which affects the Wnt/beta-catenin signaling pathway (77). These genetic abnormalities are not only pivotal for cancer progression but also modulate the tumor microenvironment to favor immune evasion and resistance to therapy.

TP53, the most commonly mutated gene in human cancers, plays a crucial role in DNA repair, cell cycle regulation, and apoptosis. Mutations in TP53 are associated with poor prognosis in liver cancer and can lead to an accumulation of genomic instability, making tumors more aggressive and resistant to conventional therapies (78).

Mutations in CTNNB1, which encodes beta-catenin, are prevalent in liver cancer. These mutations lead to the activation of the Wnt/beta-catenin signaling pathway, promoting cell proliferation and survival. Importantly, beta-catenin activation is linked to immune evasion mechanisms, such as the suppression of cytokine production and inhibition of T cell infiltration into the tumor microenvironment (79).

The genetic makeup of HCC significantly influences the effectiveness of immunotherapeutic approaches. Tumors with extensive mutational burdens may present a higher number of neoantigens, potentially enhancing their visibility to the immune system. However, the same mutations often enhance the expression of immune checkpoints like PD-L1, contributing to an immunosuppressive tumor milieu (80).

### 3.2 Checkpoint inhibitors

The use of drugs targeting immune checkpoints like PD-L1, PD-1, and CTLA-4, has been a significant development in the treatment of liver cancer, particularly hepatocellular carcinoma (HCC) (81). These immune checkpoint inhibitors (ICIs) are designated to inhibit cancer cells’ mechanisms to evade the immune system (82).

Nivolumab was among the first PD-1 inhibitors being utilized in HCC (83). Clinical trials have shown it effective in patients with advanced HCC, particularly in those who had previous treatment with the standard treatment sorafenib (84). The response rates in these trials varied but showed promising results, with some patients experiencing significant tumor reduction and prolonged survival (85).

Pembrolizumab has also been tested in HCC patients, especially those who did not respond to first-line therapies like sorafenib (86). Clinical trials reported moderate response rates, with a certain subset of patients achieving durable responses (87). Unfortunately, the overall effectiveness and best patient selection criteria for pembrolizumab in HCC are still areas of active research (88).

Atezolizumab has been studied in combination with bevacizumab, an anti-angiogenic agent. This combination has shown enhanced effectiveness compared to atezolizumab alone or other standard therapies in HCC (89). This combination has shown a promising response rate and satisfying survival rate in patients, leading to changes in first-line treatment recommendations for some HCC patients (90).

Ipilimumab, a CTLA4 monoclonal antibody, often used in combination with nivolumab, has shown effectiveness in HCC, particularly in patients who failed to respond to previous treatments. The combination of nivolumab and ipilimumab appears to have significant synergistic effect, leading to higher response rates compared to either drug alone (91).

While the response rates for ICIs in HCC vary, a significant number of patients have shown partial or complete responses. These drugs have also been associated with improved overall survival rates in certain patient groups (92). Importantly, ICIs tend to have a lasting effect for those who do respond, leading to longer periods of disease control (93).

Various clinical trials are focused on optimizing the utilization of ICIs in liver cancer, including determining the best combinations of drugs, the ideal sequencing of therapies, and identifying biomarkers to predict the most likely group of patients to benefit from these treatments (94).

### 3.3 Adoptive cell therapy

CAR-T cell therapy, a form of immunotherapy that genetically engineers the patients’ T cells to express a Chimeric Antigen Receptor (CAR) to be more capable of recognizing and killing cancer cells, is becoming a potential candidate as a treatment option for liver cancer, including hepatocellular carcinoma (HCC) (95). The current application of CAR-T cell therapy in liver cancer is primarily in the research and clinical trial phases (96). Various studies are focused on identifying suitable targets specific to liver carcinoma cells and engineering CAR-T cells to recognize these targets (97). These targets might include, for instance, GPC3 (glypican-3), which is often overexpressed in HCC (98). While still in the early stages, initial results from clinical trials suggest potential for CAR-T cell therapy in treating liver cancer. The field of CAR-T cell therapy for liver cancer is rapidly evolving, and future findings from ongoing basic and clinical research and trials are expected to provide more insights to the effectiveness and practical application of this therapy in liver cancer treatment.

### 3.4 Vaccine-based therapies

Therapeutic vaccines for liver cancer are an emerging area of research, focusing on stimulating the immune system itself to generate potent inhibition of cancer. These vaccines differ from traditional vaccines; instead of preventing disease, they are designed to treat existing cancer (42, 99).

Oncolytic virus vaccines attracted great attention of researchers and clinicians. The development of these vaccines involves genetically modifying viruses that selectively infect the cancer cells and kill them (100). Once the virus infects the tumor cells, it triggers an immune response not only against the virus but also against the tumor cells. This dual action helps in directly destroying the cancer cells and also in priming the immune system to recognize the cancer cells and induce cell death in the tumor (101).

Peptide-based vaccines serve as another strategy to treat cancer. These vaccines use specific peptides (short chains of amino acids) that are found on the outer membrane of cancer cells. After injecting these peptides, the immune system is trained to recognize and kill cells displaying these peptides, which, in most cases, are typically tumor cells in HCC (102).

The primary function of therapeutic vaccines in liver cancer is to boost the immune system’s capacity to identify and destroy cancer cells. They work by either introducing specific antigens associated with liver cancer into the body or by modifying existing immune cells to be more effective against cancer cells (42, 103). The general idea is to trigger specifically targeted immune response that leads to the destruction of cancer cells while sparing normal tissue. These therapeutic vaccines stand for a promising area of research in the treatment of liver cancer, offering potential benefits such as targeted therapy with fewer side effects compared to traditional treatments. However, most of these vaccines are still in clinical trials, and more research is needed for us to better understand their efficacy and safety in treating liver cancer.

### 3.5 Clinical trials

Several significant clinical trials have been conducted focusing on novel immunotherapeutic approaches for liver cancer, particularly hepatocellular carcinoma (HCC). Here’s an overview of some key trials and their preliminary results:

CheckMate 040 and 459 evaluated the efficacy of nivolumab, a PD-1 inhibitor, in advanced HCC patients. CheckMate 040 reported encouraging results in terms of overall response rate (ORR) and survival benefits in HCC patients, including those previously treated with sorafenib. CheckMate 459 compared first-line use of nivolumab with sorafenib. Although it failed to meet the pre-set primary endpoint of improved overall survival, nivolumab demonstrated a favorable safety profile (104, 105).

KEYNOTE-224, -240, and -394 focused on pembrolizumab in HCC. KEYNOTE-224 trial demonstrated encouraging results for pembrolizumab in sorafenib-treated patients. KEYNOTE-240 and KEYNOTE-394 trials aimed to confirm these findings in a larger cohort. The results demonstrated a better overall survival (OS) and progression-free survival (PFS), although the statistical significance varied (106–108).

The IMbrave150 trial, a pivotal phase III study, demonstrated significant advancements in treating advanced hepatocellular carcinoma (HCC) by combining atezolizumab, an anti-PD-L1 antibody, with bevacizumab, an anti-VEGF antibody, showcasing superior overall survival and progression-free survival compared to the standard treatment with sorafenib. This combination leverages dual mechanisms to modulate the tumor microenvironment, enhancing immune cell infiltration and activity while simultaneously inhibiting angiogenesis crucial for tumor growth. Despite its effectiveness, challenges such as therapy resistance—mediated by alternative immune pathways or adaptive resistance mechanisms within the tumor—persist, highlighting the need for predictive biomarkers to identify likely responders and optimize treatment regimens. Future directions include exploring synergies with other therapies and tailoring approaches based on comprehensive molecular profiling to overcome immunotolerance and improve outcomes in liver cancer treatment (109, 110).

Various ongoing trials have been exploring CAR-T cell therapy targeting specific antigens in liver cancer, such as GPC3 (111, 112). These trials are still in early phases, and results are awaited to understand the efficacy and, importantly, safety of CAR-T cells in HCC.

Trials are ongoing for vaccines targeting tumor antigens in liver cancer, such as AFP. Early-phase trials have shown some promise, but more intensive exploration is needed to establish their foundation in HCC treatment (103).

In addition, recent clinical trial updates from prominent oncology conferences, such as ASCO and ESMO, showed that the field of immunotherapy in liver cancer is rapidly advancing. The EMERALD-1 trial, combining durvalumab with bevacizumab and TACE, has shown encouraging results, significantly elongating PFS compared to TACE monotherapy (113). This underscores the potential of combining ICIs with locoregional therapies to enhance therapeutic outcomes. Furthermore, the LEAP-002 study highlights the effectiveness of combining lenvatinib with pembrolizumab, marking a step forward in dual-therapy regimens (114). Additionally, the innovative approach of the REVERT LIVER CANCER Phase 2 trial, exploring, a STAT3 inhibitor, as monotherapy and in combination, opens new avenues in targeting the liver’s immunosuppressive environment (115, 116). These developments reflect a growing exploration of the liver’s immune tolerance mechanisms and the potential of tailored combination therapies to overcome these barriers, offering new hope for patients battling liver cancer.

The most recent 2024 ASCO annual meeting reported updates on important clinical trials, such as KEYNOTE-224 and EMERALD-1, showing encouraging survival data (117, 118). CAR-T cell therapies showed promising efficacy, especially in heavily treated advanced HCC cases (119). Additionally, the oncolytic virus VG161 reported substantial disease control rates in refractory HCC (120). These studies emphasize the ongoing shift towards precision medicine, leveraging advanced genomic profiling and novel therapeutic combinations to improve outcomes for HCC patients. There are currently more than 70 ongoing clinical trials regarding immunotherapy in liver cancer (**Table 1**).

**Table 1:** Ongoing clinical trials of immunotherapy on liver cancer.

NCT Number	Study Type	Phase	Status	Sample Size(n)	Conditions	Outcome Measures
NCT05109052	Interventional	I/II	Withdrawn	48	HCC	Safety and Tolerability
NCT05185505	Interventional	IV	Recruiting	24	HCC	1 Acute Rejection 2 AE 3 ORR 4 atezolizumab/bevacizumab therapy 5 liver transplantation 6 necrotic tumors 7 RFS 8 OS 9 Tumor biomarkers 10 Immune Cell Biomarkers
NCT05609695	Observational	NA	Not yet recruiting	100	HCC	1 OS 2 TR 3 PFS
NCT05942560	Interventional	NA	Not yet recruiting	160	Depression, Anxiety, HCC, CBT	1 Depression symptoms 2 Anxiety symptoms 3 Quality of life score 4 Immune variables 5 OS
NCT05873244	Interventional	II	Recruiting	44	HCC	1 PFS 2 OS 3 radiological response rate 4 time-to-progression 5 AE
NCT05443230	Observational	NA	Enrolling by invitation	200	HCC, Sarcopenia	1 Short-term results 2 Long-term results
NCT05717400	Interventional	IV	Recruiting	15	HCC	1 Overall Response Rate
NCT05484908	Interventional	NA	Not yet recruiting	60	HCC, Liver Failure, Immune-Mediated Hepatitis	1 Mortality rate 2 Model for end-stage liver disease (MELD) score variation
NCT06045286	Interventional	I	Recruiting	30	Colorectal Liver Metastases	1 ORR 2 PFS 3 OS
NCT06199232	Interventional	NA	Not yet recruiting	47	Liver Metastasis Colon Cancer, Failed From Standard Treatment, MSS, ctDNA Genotype	1 PFS 2 OS 3 ORR 4 DCR 5 AE
NCT05550090	Observational	NA	Recruiting	40	Metastatic Breast Cancer in the Liver	1 Correlation between DCE-MRI parameters combined with IVIM parameters and efficacy of chemotherapy in patients with liver metastasis of breast cancer
NCT05438420	Interventional	I/II	Recruiting	120	HCC, Cervical Cancer, Esophageal Cancer, Gastric Cancer	1 AE 2 TR 3 Change in the area under curve (AUC) of Q702 and its primary metabolites
NCT06047015	Interventional	I/II	Not yet recruiting	12	Liver Metastasis Colon Cancer	1 Complications 2 Abscopal effect 3 Tumor-specific immune response 4 PFS 5 Quality of life questionnaire
NCT05677113	Interventional	II	Recruiting	115	Liver Metastases, Colorectal Cancer	1 PFS 2 Clearance of ctDNA 3 Side-effect profile of QBECO 4 Quality of recovery 5 Five-year overall survival
NCT05833126	Interventional	II	Recruiting	25	Recurrent Liver Cancer After Liver Transplantation	1 Acute graft rejection rate 2 ORR 3 OS 4 PFS 5 Time to Progression 6 SAE 7 Graft Rejection
NCT05451043	Interventional	II	Not yet recruiting	62	HCC, Biliary Tract Cancer, Pancreatic Cancer, Cholangiocarcinoma	1 Investigating and establishing the efficacy of propranolol in boosting the effects of immunotherapy 2 Feasibility of study therapy 3 Safety/tolerability 4 PFS 5 OS
NCT05039736	Interventional	II	Withdrawn	0	HCC	1 overall response rate
NCT05893056	Interventional	II	Recruiting	25	Gastric Cancer Metastatic to Liver	1 ORR 2 DOR 3 PFS 4 OS 5 DCR 6 Number of participants with treatment-related adverse events as assessed by CTCAE v5.0
NCT05169957	Interventional	I	Recruiting	18	Liver Metastases, Melanoma, Cutaneous, Melanoma, Mucosal, Melanoma, Ocular, Metastatic Melanoma	1 Percentage of patients who receive all planned radiotherapy 2 Proportion of patients who develop grade 3 or higher toxicity 3 OS 4 PFS 5 Proportion of patients with local control 6 ORR 7 BOR
NCT06117891	Observational	NA	Recruiting	300	Unresectable Hepatocellular Carcinoma	1 OS 2 Discriptive analysis 3 DOT 4 PFS 5 ORR 6 Treatment sequences post first-line AB or other IO combinations
NCT05588297	Interventional	II	Not yet recruiting	12	Colorectal Cancer Liver Metastases	1 R0 recession rate 2 Pathological complete response rate 3 TRG 4 ORR 5 EFS 6 DFS 7 OS 7 AE 8 Quality of life score
NCT05322187	Interventional	II/III	Not yet recruiting	15	HCC, Hepatoblastoma, Pediatric Cancer, Pediatric Solid Tumor, Transitional Cell Tumor	1 ORR 2 dynamic α-fetoprotein response (AFP-R) 3 AE 4 Health outcomes as assessed by the PROMIS^®^ Pediatric Scale v1.0 Global Health 7 + 2 scores at baseline
NCT05510427	Interventional	I	Withdrawn	0	HCC, Cholangiocarcinoma	1 AE 2 MTD
NCT05984511	Interventional	NA	Not yet recruiting	234	HCC, Tumor Thrombus, Hepatic Portal Vein Tumor Invasion	1 OS 2 PFS 3 ORR 4 Duration of portal patency 5 AE
NCT05653531	Interventional	NA	Withdrawn	0	Liver Biomarkers, ICI, Lung Cancer, Transaminases	1 basal ALT blood concentration in lung cancer patients treated with ICI determined
NCT05233358	Interventional	NA	Not yet recruiting	176	HCC	1 PFS 2 OS 3 To Tumor Untreatable Progression 4 ORR 5 DCR 6 DOR 7 AE
NCT05339581	Interventional	NA	Not yet recruiting	78	HCC, Liver Transplant; Complications, Portal Vein Thrombosis, Radiotherapy; Complications	1 PVTT RR/NR 2 Alpha Fetoprotein Response (AFP-R) 3 PFS 4 ORR 5 TTP 6 DOR
NCT05411133	Interventional	I	Recruiting	68	HCC, Cholangiocarcinoma, Colorectal Adenocarcinoma, Esophageal Adenocarcinoma, Gastric Cancer, Gastroesophageal Junction, Gastrointestinal Cancer, Pancreatic Cancer	1 AE 2 Amount of Cabotamig (ARB202) in plasma 3 Biochemical and physiological effects 4 Effect of Cabotamig (ARB202) on tumour
NCT05937295	Interventional	I	Recruiting	20	Fibrolamellar Hepatocellular Carcinoma	1 To assess immunogenicity in terms of induction of peptide specific T-cell responses 2 Safety and Tolerability
NCT05332496	Observational [Patient Registry]	NA	Recruiting	220	HCC	1 PFS 2 OS 3 ORR 4 DOR 5 DCR 6 AE
NCT05332821	Observational [Patient Registry]	NA	Recruiting	474	HCC	1 OS 2 PFS 3 ORR 4 DOR 5 DCR 6 AE
NCT05647954	Interventional	III	Not yet recruiting	350	Melanoma Neuroendocrine Tumors Neuroectodermal Tumors, Neoplasms Germ Cell and Embryonal Neoplasms by Histologic Type, Neoplasms Neoplasms	1 PFS 2 OS 3 ORR 4 DCR 5 DOR 6 PFS 7 OS 8 AE
NCT05810402	Interventional	NA	Not yet recruiting	60	HCC, ICI, Liquid Biopsy	1 Percentage of patients with CTCs-PD-L1+ by CellSearch^®^ technique 2 OS 3 PFS
NCT06031480	Interventional	II	Not yet recruiting	55	HCC	1 ORR
NCT04430452	Interventional	II	Recruiting	21	HCC	1 ORR 2 AE 3 PFS 4 DOR 5 OS
NCT06040177	Interventional	I/II	Recruiting	30	HCC Non-resectable, ICI, Portal Vein Tumor Thrombus	1 ORR 2 PFS 3 DCR 4 DOR 5 OS
NCT06205706	Interventional	I/II	Recruiting	104	HCC, Non Small Cell Lung Cancer, Solid Tumors	1 AE 2 SAE 3 Frequency of dose interruptions and dose reductions 4 DLT
NCT05278195	Observational	NA	Recruiting	300	HCC	1 OS 2 Specificity 3 Sensitivity 4 The area under curve (AUC) of Receiver Operating Characteristic (ROC) curves of the radiomics artificial intelligence mode 5 Accuracy
NCT05406466	Interventional	II	Recruiting	25	Melanoma	1 ORR 2 DCR 3 DOR 4 TTR 5 PFS 6 OS 7 AE
NCT05070247	Interventional	I/II	Recruiting	313	HCC, Breast Cancer, Esophageal Cancer, Gastric Cancer, Kidney Cancer, Mesothelioma, Nasopharyngeal Cancer, Non-small Cell Lung Cancer (NSCLC), Non-squamous, Pancreatic Cancer, Squamous Cell Cancer of Head and Neck (SCCHN)	1 Dose Escalation 2 Dose Expansion: Overall Response Rate (ORR) 3 DCR 4 DOR 5 TTR 6 PFS 7 OS 8 AE
NCT05665348	Interventional	II/III	Not yet recruiting	574	HCC, Metastatic Tumor	1 Objective response of treatment 2 OS 3 PFS 4 OR
NCT05879328	Observational	NA	Recruiting	12	HCC	1 RFS 2 TR 3 Complication rate 4 OS 5 Patients’ reported outcomes (PROs) 6 Comparison with historical series
NCT04777851	Interventional	III	Recruiting	496	HCC	1 PFS 2 OS 3 ORR 4 Time to unTACEable Progression (TTUP) 5 DOR
NCT04965714	Interventional	II	Withdrawn	0	Resectable HCC	1 AE 2 Rate of pathologic complete response 3 Necrosis of tumors 4 TTP 5 RFS 6 OS
NCT06041477	Interventional	III	Recruiting	540	HCC, Chemotherapeutic Toxicity, Chemotherapy Effect	1 PFS 2 OS 3 ORR 4 DCR 5 CRR 6 Safety profiles of all participants
NCT05897268	Interventional	II	Recruiting	25	HCC	1 ORR 2 PFS 3 OS 4 DOR 5 DCR 6 ORR 7 PFS 8 OS 9 AE
NCT05096715	Interventional	I	Not yet recruiting	20	Unresectable HCC	1 Dose Limiting Toxicity Rate 2 PFS 3 OS 4 In-field response rate 5 Change in Child-Pugh Score 6 Out of field response rate
NCT05092373	Interventional	I	Recruiting	36	too much	1 To assess the safety and tolerability of TTF, including the maximum tolerated dose (MTD) 2 ORR 3 PFS 4 OS
NCT05578430	Interventional	II	Not yet recruiting	54	Resectable HCC	1 MPR 2 RFS 3 ORR 4 AE
NCT05044676	Observational	NA	Recruiting	120	HCC	1 OS
NCT05516628	Interventional	II	Not yet recruiting	30	HCC	1 RFS 2 TTR 3 RFS 4 OS
NCT06218511	Interventional	I	Recruiting	10	HCC	1 DFS 2 PFS 3 OS 4 AE
NCT05625893	Interventional	II	Recruiting	63	HCC, Portal Vein Thrombosis	1 PFS 2 AE 3 OS 4 Time-to-progression 5 ORR 6 DCR 7 Local tumor progression rate
NCT04965454	Interventional	II	Recruiting	80	HCC Non-resectable	1 ORR 2 DCR
NCT05337137	Interventional	I/II	Recruiting	162	HCC	1 DLT 2 ORR 3 PFS
NCT06133062	Interventional	II	Recruiting	45	HCC Non-resectable	1 PFS 2 LC 3 TTP 4 ORR 5 OS 6 AE
NCT05537402	Interventional	II	Recruiting	204	HCC	1 PFS 2 ORR 3 OS
NCT05717738	Observational	NA	Recruiting	300	HCC Non-resectable	1 Response Rate measured by mRECIST criteria 2 Number of Patients Amendable to Curative Surgical Interventions 3 TTP 4 PFS 5 OS 6 Pathological response 7 DCR 8 Quality of Life (QoL)
NCT05168163	Interventional	II	Recruiting	122	HCC	1 OS 2 PFS 3 ORR 4 DOR 5 AE
NCT05620771	Interventional	II	Recruiting	84	HCC	1 PFS 2 TTP 23 ORR 4 DOR 5 CBR 6 OS 7 AE
NCT05389527	Interventional	II	Active, not recruiting	43	HCC	1 MPR 2 PCR 3 Pathologic complete response (pCR) 4 ORR 5 R0 resection rate 6 DFS 7 OS 8 AE
NCT05488522	Interventional	I	Recruiting	18	HCC	1 Primary Objective 2 Secondary Objective 3 OS 4 PFS
NCT05101629	Interventional	II	Active, not recruiting	32	HCC	1 ORR 2 OS 3 Safety and toxicity
NCT05199285	Interventional	II	Recruiting	40	HCC	1 ORR 2 OS 3 PFS 4 Disease control 5 AE
NCT05822752	Interventional	II	Recruiting	120	HCC	1 BOR 2 DOR 3 PFS 4 OS
NCT05269381	Interventional	I	Recruiting	36	too much	1 AE 2 The number and percentage of participants who completed the sequencing with satisfactory data quality registration and identified at least 10 actionable peptides, meet the eligibility criteria for registration, and able to initiate vaccine production 3 Immunogenicity responders
NCT05327738	Interventional	II	Withdrawn	0	HCC	1 Proportion of progression-free participants 2 ORR 3 DCR 4 TTP 5 PFS 6 OS 7 Incidence of grade >= 3 adverse events
NCT05377034	Interventional	II	Recruiting	176	Locally Advanced Hepatocellular Carcinoma	1 BOR 2 DOR 3 TOR 4 PFS 5 OS
NCT05286320	Interventional	I/II	Not yet recruiting	27	Unresectable Hepatocellular Carcinoma, Lenvatinib, Pembrolizumab, Stereotactic Body Radiotherapy	1 safety rate 2 ORR 3 PFS 4 OS 5 Immune biomarkers
NCT06024252	Observational	NA	Not yet recruiting	200	HCC	1 OS 2 PFS 3 ORR 4 One-year survival rate 5 Immune-TACE PFS 6 DCR 7 Treatment pattern
NCT05448677	Interventional	II	Recruiting	196	HCC	1 PFS 2 ORR
NCT05223816	Interventional	II	Recruiting	97	HCC, Intrahepatic Cholangiocarcinoma	1 Safety in Cohort1 2 ORR 3 PFS
NCT05797805	Interventional	I/II	Recruiting	108	Advanced Hepatocellular Carcinoma	1 AE 2 DLT 3 Evaluate efficacy of tegavivint as a single agent
NCT05776875	Interventional	II	Recruiting	24	HCC	1 AE 2 Response rate 3 Time to progression 4 Time to TACE progression (TTTP) 5 Time to untaceable progression
NCT05908786	Interventional	I/II	Recruiting	150	HCC	1 MPR 2 PCR 3 Relapse-Free Survival (RFS) 4 Event-Free Survival (EFS) 5 OS
NCT05396937	Interventional	II	Recruiting	42	HCC	1 ORR 2 Duration of Objective Response (DoR) 3 DCR 4 TTP 5 PFS 6 OS
NCT05903456	Interventional	II	Not yet recruiting	20	HCC	1 ORR 2 PFS 3 OS 4 DCR 5 Disease Control Rate 6 DOR 7 AE
NCT06066333	Interventional	II	Recruiting	12	ACC, Adrenocortical Carcinoma, Metastatic Adrenocortical Carcinoma	1 AE

## 4 Strategies to overcome the tolerogenic microenvironment

### 4.1 Combination therapies

The rationale for combining other therapies with immunotherapy in liver cancer treatment stems from several key factors. Firstly, immunotherapies alone might not be fully effective due to the liver’s immune-tolerant nature and the complex tumor microenvironment in conditions like hepatocellular carcinoma (HCC) (121). Combining these therapies can enhance overall efficacy and overcome the resistance that often develops against single-treatment modalities. Moreover, liver cancer involves various biological pathways, and a combination approach allows for a more comprehensive targeting of the disease. Such combinations can also produce synergistic effects; for instance, certain chemotherapy induces immunogenic cell death, potentially enhancing the immune system’s recognition and attack on tumor cells (122). Additionally, this strategy might allow for lower dosages of each treatment, potentially reducing side effects while maintaining or improving efficacy. Finally, certain therapies can modify the tumor’s immune microenvironment, which becomes more susceptible to an immune attack, thus supporting the effectiveness of immunotherapy. This multi-modal approach is central to current research in liver cancer, aiming to significantly improve patient outcomes.

Combining Immunotherapy with Chemotherapy leverages the direct tumor-killing effect of chemotherapy and the immune-modulating properties of immunotherapy. Chemotherapy can release cancer antigens, making tumor cells easier to recognize by the immune system, while immunotherapy can strengthen the immune response against these exposed antigens.

Another important strategy is to combine immunotherapy and targeted therapy. Targeted therapies work by acting on specific molecular targets related to cancer. When combined with immunotherapy, these therapies can disrupt cancer cell mechanisms that suppress the antitumor immunity, enhancing the function of immunotherapeutic agents. Overcoming resistance to immune checkpoint inhibitors (ICIs) is significantly enhanced by incorporating anti-angiogenic drugs, which target the vascular endothelial growth factor (VEGF) pathway (123). These drugs aid in normalizing the tumor’s abnormal vasculature, which not only improves blood flow and oxygenation within the tumor, thereby reducing hypoxia, but also facilitates the infiltration of effector T cells into the tumor microenvironment (124). This process enhances the immune system’s capacity to target and destroy tumor cells. Additionally, anti-angiogenic therapies help reduce the recruitment of immunosuppressive cells such as regulatory T cells (Tregs) and myeloid-derived suppressor cells (MDSCs) to the tumor site and alter immune-related signaling, including the modulation of PD-L1 expression on tumor and immune cells (125). The combination of the immune checkpoint inhibitor atezolizumab (PD-L1 inhibitor) with bevacizumab (VEGF inhibitor) has become the recommended first-line systemic treatment for advanced HCC (109). A case study reported the successful treatment of brain metastasis in intrahepatic cholangiocarcinoma with a combination of the PD-1 inhibitor camrelizumab and a multi-kinase inhibitor lenvatinib. The patient showed a complete response (CR) and a PFS of 17.5 months without serious side effects, suggesting the potential of this combination therapy (126).

Dual Immune Checkpoint Inhibition is another widely-used approach. Using two different immune checkpoint inhibitors can have a synergistic effect. This combination can enhance T-cell activation and more effectively attack cancer cells than single-agent therapy (127). In the HIMALAYA study, Tremelimumab and durvalumab show potential in treating unresectable, advanced liver cancer, offering a new choice for inflammation-driven cancer (128).

Techniques like radiofrequency ablation (RFA) or transarterial chemoembolization (TACE) can also be combined with immunotherapy (129, 130). These local treatments can increase antigen presentation and inflammation, potentially making immunotherapy more effective (131).

In addition, therapeutic cancer vaccines can be combined with immunotherapies to enhance the immune response specifically against liver cancer cells (132).

These combination therapies aim to capitalize on the strengths of each treatment modality, aiming for a more robust and targeted attack on liver cancer cells (12). Clinical trials are still being carried out to find out the most effective combinations and protocols (42).

Preclinical studies also gave sight to novel strategies to enhance the effect of immunotherapy. For instance, a recent study reported that antitumor immunity can be enhanced by targeting cGas/STING pathway (133). Targeting fibrinogen-like protein 1 can also enhance immunotherapy in hepatocellular carcinoma (134).

### 4.2 Personalized medicine by immune classification

Personalized approaches, including the development of biomarkers for the prediction of immunotherapy outcome, are increasingly important in liver cancer treatment, allowing for more targeted and effective treatments (135).

The immune microenvironment of liver cancer can be classified based on molecular features and immunogenicity into distinct types, reflecting the heterogeneity and complexity of tumor immune interactions (22). Based on the differentiated infiltration of the cytotoxic immune cells, primary liver cancers are categorized into inflamed tumors, which are immunologically active, and non-inflamed tumors, which are immunologically inactive (136). Recent studies further identified four immune subclasses of liver cancer according to their immunosuppression mechanisms and genomic alterations, namely, 1) Tumor-associated macrophage (TAM): This subclass shows increased levels of extracellular matrix genes, and is associated with poor survival (137). 2) CTNNB1: characterized by CTNNB1 mutations (138). 3) Cytolytic activity (CYT): Represents inflamed tumors with high cytolytic activity (139). 4) Regulatory T cell (Treg): Also represents inflamed tumors but with increased presence of Treg cells (140). The TAM and CTNNB1 subclasses are seen as non-inflamed, while the CYT and Treg subclasses represent inflamed tumors (141). Further classification based on immunogenomic features has led to the identification of three HCC subtypes based on immune characteristics: immunity high (referred as Immunity_H), medium (Immunity_M), and low (Immunity_L). This classification is effectively predictive of patient prognosis, with the Immunity_H subtype indicating a better survival rate due to higher immune and stromal scores (85, 89).

The classifications of the liver cancer immune microenvironment based on molecular features and immunogenicity enabled personalized therapeutic strategies (142). Understanding the specific immune subclass of a liver tumor allows for selecting patients more likely to respond to immunotherapies, as well as developing targeted therapies (143). For instance, patients with inflamed tumors might have a higher responding rate to Immunotherapies due to the presence of active immune cells in the tumor (144). Tumors in the TAM subclass might benefit from therapies targeting TAMs or the extracellular matrix to reverse immunosuppression and enhance immune activity against the tumor (145). Moreover, the identification of Immunity subclasses can serve as predictive biomarkers for patient prognosis. Patients with the Immunity_H subtype, characterized by higher stromal and immune scores, have a better survival rate, indicating that these patients might respond better to immunotherapies (137, 146). This information is crucial for clinical decision-making and modifying treatment approaches based on individual tumor features (99, 147). By identifying the specific mechanisms of immune resistance in different liver cancer subclasses, therapies can be tailored to counteract these mechanisms (148). For instance, if a tumor employs specific checkpoint pathways to evade immune surveillance, checkpoint inhibitors targeting those pathways can be used (149).

Strategies based on the immune classification enables a more precise and personalized approach to liver cancer treatment (150). By understanding the molecular and immunological landscape of individual tumors, treatments can be tailored to target specific pathways and immune cells involved in tumor progression, leading to more effective and less toxic treatment options (151).

## 5 Conclusion and future directions

**Figure 3:**
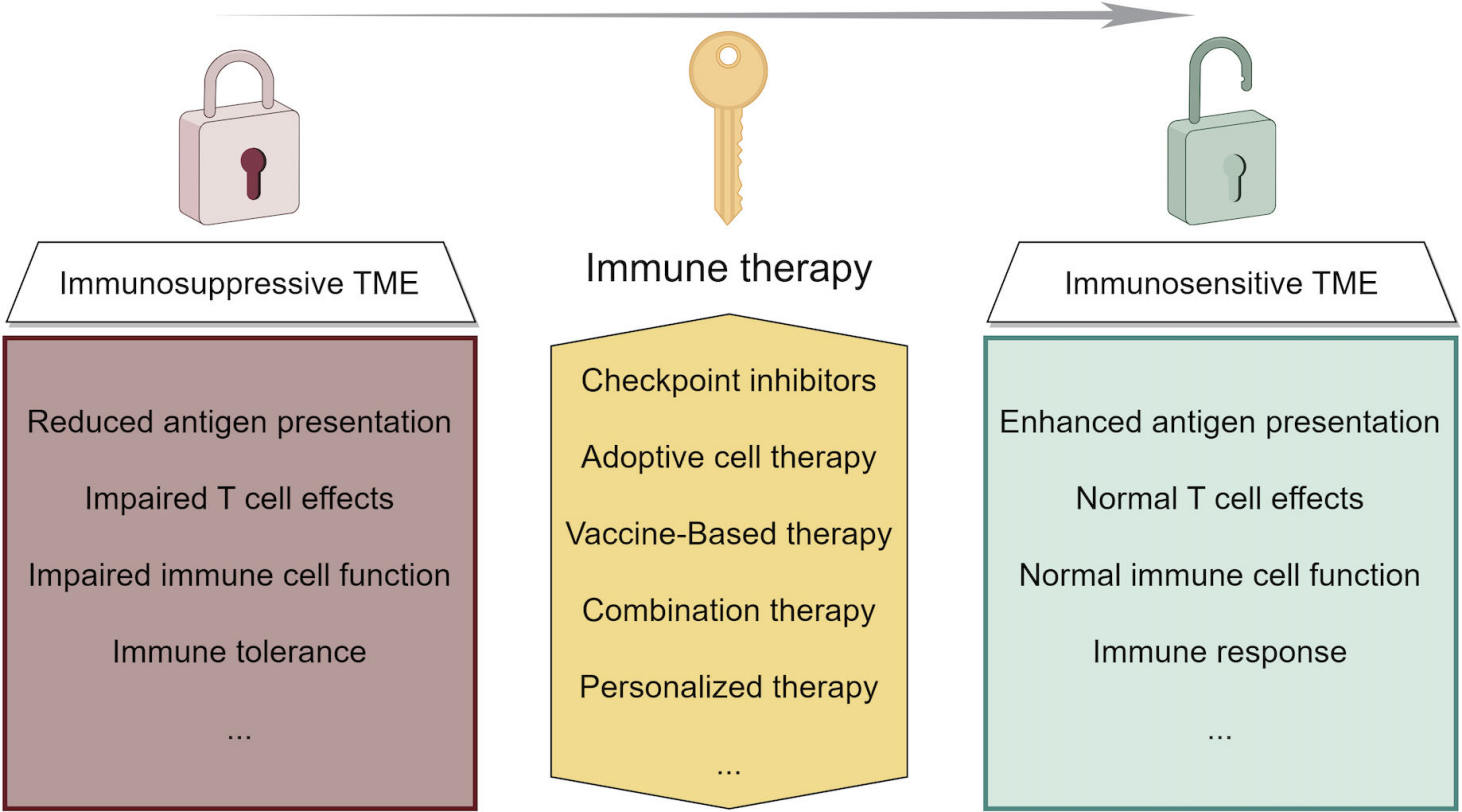
Strategies of immunotherapy in liver cancer and their function of modifying the tumor microenvironment.

Recent advances in liver cancer immunotherapy, particularly in HCC, have highlighted several key findings, including the efficacy of novel ICIs, the potential of combination therapies, and the importance of personalized approaches based on biomarkers (152). These developments suggest a future where liver cancer treatments are more tailored and effective (153). The focus is shifting toward understanding the liver’s unique immune environment and developing therapies to overcome its inherent challenges (**Figure 3**). The future outlook for liver cancer immunotherapy is promising, with ongoing research aimed at improving response rates and patient outcomes through more targeted, personalized treatment. Future research in immunotherapy for liver cancer should focus on combination therapies that merge different immunotherapeutic strategies or pair them with traditional treatments to overcome the immunosuppressive tumor microenvironment. Personalized immunotherapies based on genomic profiling, alongside the development of predictive biomarkers, could tailor treatments to individual patient profiles for improved efficacy. Targeting regulatory T cells, exploring new immunotherapeutic targets, and enhancing T cell responsiveness within the suppressive liver environment are promising directions. Studies should also address inherent or acquired resistance mechanisms to optimize therapeutic outcomes. Innovative clinical trial designs that incorporate dynamic endpoints and real-time biomarker analysis can expedite the advancement of effective treatments. An integrative approach combining genomic, proteomic, and clinical data might offer a comprehensive understanding of disease mechanisms and therapy interactions, paving the way for breakthroughs in liver cancer immunotherapy.
